# Drug Resistance Mutations for Surveillance of Transmitted HIV-1 Drug-Resistance: 2009 Update

**DOI:** 10.1371/journal.pone.0004724

**Published:** 2009-03-06

**Authors:** Diane E. Bennett, Ricardo J. Camacho, Dan Otelea, Daniel R. Kuritzkes, Hervé Fleury, Mark Kiuchi, Walid Heneine, Rami Kantor, Michael R. Jordan, Jonathan M. Schapiro, Anne-Mieke Vandamme, Paul Sandstrom, Charles A. B. Boucher, David van de Vijver, Soo-Yon Rhee, Tommy F. Liu, Deenan Pillay, Robert W. Shafer

**Affiliations:** 1 World Health Organization, Geneva, Switzerland; 2 Molecular Biology Laboratory, Centro Hospitalar de Lisboa Ocidental, Lisbon, Portugal; 3 Molecular Diagnostics, “Prof. Dr. Matei Bals” Institute for Infectious Diseases, Bucharest, Romania; 4 Brigham and Women's Hospital Harvard Medical School, Boston, Massachusetts, United States of America; 5 Laboratoire de Virologie EA 2968, Université de Bordeaux, Bordeaux, France; 6 Division of Infectious Diseases, Stanford University, Stanford, California, United States of America; 7 Centers for Disease Control and Prevention, Atlanta, Georgia, United States of America; 8 Division of Infectious Diseases, Brown University, Providence, Rhode Island, United States of America; 9 Tufts University School of Medicine, Boston, Massachusetts, United States of America; 10 Rega Institute for Medical Research, Katholieke Universiteit Leuven, Leuven, Belgium; 11 Centre for Infectious Disease Prevention and Control, Public Health Agency of Canada, Ottowa, Ontario, Canada; 12 Department of Virology, Erasmus MC, University Medical Center Rotterdam, Rotterdam, the Netherlands; 13 Department of Medical Microbiology, University Medical Center Utrecht, Utrecht University, Utrecht, the Netherlands; 14 Centre for Virology, Division of Infection and Immunity, University College London and Centre for Infections, Health Protection Agency, London, United Kingdom; University of California San Francisco, United States of America

## Abstract

Programs that monitor local, national, and regional levels of transmitted HIV-1 drug resistance inform treatment guidelines and provide feedback on the success of HIV-1 treatment and prevention programs. To accurately compare transmitted drug resistance rates across geographic regions and times, the World Health Organization has recommended the adoption of a consensus genotypic definition of transmitted HIV-1 drug resistance. In January 2007, we outlined criteria for developing a list of mutations for drug-resistance surveillance and compiled a list of 80 RT and protease mutations meeting these criteria (surveillance drug resistance mutations; SDRMs). Since January 2007, several new drugs have been approved and several new drug-resistance mutations have been identified. In this paper, we follow the same procedures described previously to develop an updated list of SDRMs that are likely to be useful for ongoing and future studies of transmitted drug resistance. The updated SDRM list has 93 mutations including 34 NRTI-resistance mutations at 15 RT positions, 19 NNRTI-resistance mutations at 10 RT positions, and 40 PI-resistance mutations at 18 protease positions.

## Introduction

The worldwide effort to improve treatment outcomes and reduce transmission of HIV through optimal delivery of ART and HIV prevention programmes must be coordinated with and enlightened by ongoing national, regional, and global evaluations of HIV drug resistance. One essential element in the global evaluation is population-based surveillance of transmitted HIV drug resistance in recently infected individuals. As HIV drug resistance surveillance programs are underway in many countries and regions, it has become essential to develop a standard list of mutations to characterize the epidemiology of transmitted drug resistance [1], [2], [3], [4], [5]. Only with a standard list of mutations is it possible to compare the prevalence of transmitted resistance from different times and regions and facilitate meta-analyses of surveillance data collected by different groups at different times. Compiling such a standard list, however, is not simple because of the rapidly changing field of ARV therapy and the large numbers of mutations associated with ARV drug resistance [6], [7].

In 2007, we outlined four criteria for identifying surveillance drug-resistance mutations (SDRMs) and used these criteria to create a provisional list of SDRMs [6]. The first criterion was that SDRMs should be recognized as causing or contributing to drug resistance – defined as being present on three or more of five expert lists of drug resistance mutations. The second criterion was that mutations should be non-polymorphic and should not occur at highly polymorphic positions. The third criterion was that the mutation list had to be applicable to the eight most common HIV-1 subtypes. The fourth criterion was that the list should be parsimonious, excluding mutations resulting exceedingly rarely from drug pressure.

Since the 2007 list was published, new drug-resistance mutations have been identified including mutations arising from the increased use of non-thymidine-analog containing regimens, the expanded use of two new protease inhibitors (PIs), and the recent approval of a new non-nucleoside RT inhibitor (NNRTI). The number of sequences from ARV-naïve persons infected with subtype B and non-B HIV-1 viruses in our analysis dataset has approximately doubled since the 2007 publication, increasing the confidence with which nonpolymorphic mutations can be identified. In this paper, we followed the same steps used to create the 2007 mutation list.

## Methods

### Identification of mutations causing or contributing to drug resistance

Mutations that were present on three or more of the following five expert lists – ANRS drug resistance interpretation algorithm (2008.07), HIVdb drug resistance interpretation algorithm (4.3.7), IAS-USA Mutations Associated With Drug Resistance (March/April 2008), Los Alamos National Laboratories HIV Sequence database (2007), or Rega Institute Drug Resistance Interpretation Algorithm (7.1.1) – were considered to be recognized as causing or contributing to drug resistance. The complete list of mutations associated with each of these lists can be found on the Surveillance Drug Resistance Mutation (SDRM) worksheet (http://hivdb.stanford.edu/cgi-bin/AgMutPrev.cgi).

### Identification of nonpolymorphic mutations and mutations not occurring at highly polymorphic positions

Some drug resistance mutations occur commonly in the absence of drug selective pressure, these polymorphic drug-resistance mutations should not be used for surveillance of transmitted drug resistance because they could lead to falsely elevated estimates of transmitted resistance. For the purposes of generating a nonpolymorphic list of drug resistance mutations, we defined nonpolymorphic mutations to be mutations present at a frequency ≤0.5% in ARV-naïve individuals infected with subtypes for which >1,000 sequences were available in our dataset and at levels >0.5% in no more than one subtype for which fewer than 1,000 sequences were available. Nonpolymorphic mutations occurring at polymorphic positions, defined as positions with mutations occurring at >1% in any subtype, were generally excluded. Exceptions were made for major mutations that directly contribute to causing resistance.

### Assignment of HIV-1 subtype

A set of 100 reference sequences was compiled by combining 65 representative group M sequences curated by the Los Alamos Sequence Database and an additional 35 samples added so that the dataset would include three or more divergent reference sequences for each pure subtype and many of the most common CRFs. Neighbor joining trees were created from an alignment of each sequence with the 100 reference sequences. Sequences clustering within clades formed by subtypes A, B, C, D, F, G, H, J, and K, and CRF01_AE and CRF02_AG sequences were assigned to that clade. Sequences grouping within clades CRF_03 to CRF_19 were assigned to that clade unless the region spanned by the CRF mapped onto one of the pure subtypes or CRF01_AE or CRF02_AG, in which case the sequence was assigned to one of these. Sequences that were not within a clade were assigned to the subtype or CRF of the closest node. For 85.4% of ARV-naive sequences, the subtype matched the STAR program subtype [8] subtype; for 4.5% of sequences, the subtype differed from the STAR subtype, and for 10.1% of isolates, the STAR program did not provide a definitive result. For the purposes of this study, we analyzed mutation prevalence rates only within subtypes A, B, C, D, F, G, CRF01_AE, and CRF02_AG. CRF sequences and non-CRF recombinants that clustered with one of these eight subtypes within protease and/or RT were also included.

### Exclusion of rare mutations

The fourth consideration in creating the SDRM list was that it should be as parsimonious as possible without sacrificing sensitivity. To accomplish this, we excluded exceedingly rare drug-resistance mutations defined as those mutations present at a frequency below 0.5% among treated individuals in the subtype having the highest prevalence of that mutation. Because the number of isolates from treated persons for some subtypes was low, we also required that the mutations be present in sequences from at least two different persons with the subtype having the highest prevalence of that mutation.

### Analysis and review

We identified mutations present on three or more of the selected lists and analyzed publicly available RT and PI sequences reported as being from drug-naive individuals, within subtypes A, B, C, D, F, G, CRF01_AE, and CRF02_AG, for the frequency of each mutation by subtype. To reduce the influence of transmitted resistance on the identification of non-polymorphic mutations among the sequences in our dataset we excluded treatment-naïve individuals from studies of primarily infected persons in regions with high rates of transmitted resistance and excluded sequences with two or more mutations from the 2007 SDRM list based on the premise that such sequences were likely to have resulted from previous selective drug pressure. The next phase of the analysis included only mutations that met the criteria for non-polymorphism.

For each of the mutations that met our criteria for non-polymorphism and for non-occurrence at a highly polymorphic position in the previous analysis, we examined publicly available sequences for the frequency of each mutation among individuals reported to be treated with the relevant drug class. This analysis was also performed separately for each subtype. The mutations that met the criterion for rarity were excluded from the list.

We reached the final list through review of the results of the analysis by a panel comprising the authors of this paper. Some mutations that occurred as low-level polymorphisms among several subtypes were further excluded for parsimony. These are described in the results section.

Finally, because the mutations on the resulting 2009 SDRM list included those occurring in sequences of untreated individuals at a frequency of 0.1% to 0.5% (and for three mutations at a frequency of >0.5%) in one or more subtypes, we examined the frequencies of sequences with one or more mutations on the 2009 SDRM list for each ARV drug class and each subtype among the sequences in the dataset from untreated persons.

## Results

### Identification of drug-resistance mutations

The SDRM worksheet on the HIV Drug Resistance Database (http://hivdb.stanford.edu/cgi-bin/AgMutPrev.cgi) shows all of the mutations present on the ANRS, HIVdb, IAS-USA, Los Alamos, and Rega algorithm lists. Overall, 75 mutations were on five lists including 17 NRTI, 18 NNRTI, and 40 PI-associated mutations; 43 mutations were on four lists including 11 NRTI, 8 NNRTI, and 24 PI-associated mutations; and 42 mutations appeared on three lists including 17 NRTI, 11 NNRTI, and 14 PI-associated mutations.

### Sequences from untreated Individuals

RT sequences from 11,586 RT inhibitor-naïve individuals and protease sequences from 15,220 PI-naïve individuals were publicly available and met the criteria to be included the analysis dataset (Table 1). More than 50% of both the RT and protease sequences were from non-subtype B viruses. The dataset contains more than double the number of protease and RT sequences available from untreated persons infected with viruses from both subtype B and non-B subtypes compared with the number of sequences used to generate the 2007 SDRM list [6]. RT and protease sequences from more than 1,000 individuals were available for subtype A, subtype B, subtype C, and CRF02_AG. For 70.1% of the sequences, both the protease and RT gene were sequenced; for 17.9%, only the RT gene was sequenced; and for 12.0%, only the protease gene was sequenced.

**Table 1:** Numbers of Reverse Transcriptase Inhibitor (RTI) and Protease Inhibitor (PI)-Naïve Persons from Whom Publicly Available Sequences Were Available for Analysis in 2006 and 2008.

Subtype	RTI-Naïve Persons			PI-Naïve Persons		
	2006	2008	% Δ	2006	2008	% Δ
B	2,240	5,672	+153	3,704	7,439	+101
***Non-B Subtypes***						
A	499	1,305	+162	686	1,528	+123
AE	635	770	+21	684	902	+32
AG	484	1,035	+114	811	1,437	+77
C	915	2,020	+121	1025	2,182	+113
D	192	320	+69	355	515	+45
F	137	265	+93	180	598	+232
G	145	403	+178	270	619	+129
Non-B Total	3,007	6,118	+105	4011	7,781	+94

### Sequences from treated individuals

RT sequences from 14,622 RT inhibitor-treated individuals and protease sequences from 7,819 PI-treated individuals were publicly available and met the criteria to be included the analysis dataset. The relative proportions of non-B sequences compared with subtype B sequences were considerably lower among treated individuals than among untreated individuals. The number of RT inhibitor-treated individuals with non-B subtypes was 3,680, approximately 1/3 the number of individuals (10,942) with subtype B sequences. The number of sequences from RT inhibitor-treated persons with non-B viruses ranged from 248 for subtype D to 1,063 for subtype C. The number of PI-treated individuals with non-B sequences was 1,168, approximately 1/5 the number of treated individuals (6,651) with subtype B sequences. The number of isolates from PI-treated individuals with non-B viruses ranged from 61 for CRF01_AE to 307 for subtype F. Non-B sequences made up 25% of the analyzable RT sequences and 15% of the analyzable PI sequences.

### NRTI-Associated SDRMs

There were 39 nonpolymorphic NRTI-associated drug resistance mutations present on three or more expert lists (http://hivdb.stanford.edu/cgi-bin/AgMutPrev.cgi). Sixteen mutations were on five lists; nine mutations were on four lists; and 14 mutations were on three lists. Among these mutations, two occurred at a frequency of >0.5% in subtype D infected individuals: M41L in four (1.2%) and M184V in two (0.6%) of 324 individuals. Examination of the sequences with these mutations displayed no evidence for sequence artifact or epidemiological clustering.

Six nonpolymorphic NRTI-resistance mutations including E44A, D67E, T69N, K70E, L74I, and K219N were not present on the 2007 SDRM list. We added four of these mutations – D67E, K70E, L74I, and K219N – to the updated SDRM list (Table 2). Two mutations (E44A and T69N) were not added because they occur at polymorphic positions. Moreover, T69N occurred in 0.4%, 0.4%, and 0.5% of subtypes B, F, and CRF01_AE, respectively.

**Table 2:** Nucleoside RT Inhibitor Surveillance Drug Resistance Mutation (SDRM) List: 34 Mutations at 15 Positions

Position	AA	Lists	New	A (%)	AE (%)	AG (%)	B (%)	C (%)	D (%)	F (%)	G (%)	No Rx (Max %)	Max Rx (%)
***Number of individuals:***				**1,305**	**770**	**1,035**	**5,672**	**2,020**	**324**	**265**	**403**	**11,586**	**14,621**
41	L	5		0	0.3	0.1	0.3	0	**1.2**	0	0	1.2	39
65	R	5		0.1	0	0	0	0.2	0	0	0	0.2	6.5
67	N	5		0	0	0	0	0	0	0	0	0	44
	G	4		0	0	0.1	0.2	0.1	0	0	0	0.2	1.6
	E	3	√	0	0	0	0	0	0	0	0	0	1.7
69	D	4		0.1	0	0	0	0	0	0	0	0.1	7.1
	ins	5		0	0	0	0	0	0	0	0	0	1.2
70	R	5		0	0.3	0	0.2	0	0	0	0	0.3	29
	E	5	√	0	0	0	0	0	0	0	0	0	1.6
74	V	5		0	0	0	0	0	0	0	0.2	0.2	7.5
	I	4	√	0	0	0	0	0	0	0	0	0	3.3
75	M	4		0	0.5	0	0	0	0	0	0	0.5	3.8
	T	4		0	0	0	0	0	0	0	0	0	2.7
	A	3		0	0	0	0	0.1	0	0	0	0.1	0.8
	S	3		0	0	0	0	0	0	0	0	0	1.4
77	L	3		0	0.3	0	0.1	0	0	0	0	0.3	2.0
115	F	5		0	0	0	0	0	0	0	0	0	1.4
116	Y	3		0	0.1	0	0	0	0	0	0	0.1	3.3
151	M	5		0	0	0	0	0	0	0	0	0	5.2
184	V	5		0.2	0.1	0.2	0.2	0.1	**0.6**	0	0	0.6	61
	I	5		0	0.3	0.1	0	0	0.3	0	0	0.3	2.8
210	W	5		0	0	0	0	0	0	0	0	0	25
215	Y	5		0.2	0	0	0	0	0	0	0	0.2	38
	F	5		0.1	0	0	0	0	0	0	0	0.1	22
	I	3		0.2	0.1	0	0	0	0	0	0	0.2	5.7
	S	3		0	0.1	0.1	0.4	0	0	0.4	0	0.4	0.6
	C	3		0	0	0	0.2	0	0	0	0	0.2	3.1
	D	3		0	0	0	0.4	0	0.3	0	0	0.4	0.8
	V	3		0	0	0	0	0	0	0	0	0	1.0
	E	3		0	0	0	0.1	0	0	0	0	0.1	2.1
219	Q	5		0.2	0.4	0.4	0	0	0	0	0	0.4	25
	E	5		0	0	0	0	0	0	0	0	0	9.4
	N	4	√	0.1	0.4	0.2	0.1	0	0	0	0	0.4	2.5
	R	4		0.2	0	0.1	0.1	0.1	0	0	0	0.1	2.2
***Sum of Prevalences:***				**1.4**	**2.9**	**1.3**	**2.3**	**0.6**	**2.4**	**0.4**	**0.2**		

**Abbreviations:** Pos – amino acid position; AA – amino acid difference from consensus B; Lists – Number of mutation lists with the mutation; New – mutations not present on the 2007 SDRM list; No Rx – highest prevalence in untreated persons in any of the 8 listed subtypes; Max Rx – Prevalence of the mutation in the subtype with the highest prevalence of the mutation provided the mutation is present in viruses from two or more individuals. Underlined bold mutations are those with a prevalence >0.5% that are nonetheless included because the >0.5% prevalence is in only one subtype with fewer than 1000 sequences available for analysis.

Although they occur at polymorphic positions, we included in the list the NRTI mutations T69D, the T69 insertions, and V75T/M/A/S, because of their substantial contribution to resistance to commonly-used NRTIs.

Several known NRTI-resistance mutations were excluded from consideration as an SDRM: (i) K65N is a recently described rare NRTI-resistance mutation, which was present on two expert lists and which appears to have a phenotypic effect similar to K65R [9], [10]. However, it has been reported in only six NRTI-experienced and nine NRTI-naïve individuals, amd its prevalence was less than 0.1% among sequences from treated individuals in any subtype. (ii) A62V is an accessory NRTI-resistance mutation which is nonpolymorphic except for its presence in 16% of subtype A viruses due to a founder effect within the intravenous drug user epidemic in Eastern Europe [11]; (iii) E44D and V118I were not included because they are polymorphic in multiple subtypes; (iv) Deletions at codon 67 were deleted from the 2007 list because their highest frequency among treated individuals of any subtype was 0.1%; and (v) K70G is a mutation which has an effect similar to K70E but was present on only two expert lists and was present at no more than 0.3% of treated individuals with any subtype.

Table 2 shows the updated list of 34 NRTI SDRMs at 15 RT positions. The proportion of reportedly drug-naive individuals in the dataset having one or more NRTI-associated SDRMs for each of the eight subtypes is as follows: 0.2% in subtype G, 0.4% in subtype F, 0.6% in subtype C, 1.3% in CRF02_AG, 1.4% in subtype A, 2.3% in subtype B, 2.4% in subtype D, and 2.9% in CRF01_AE.

### NNRTI SDRMs

There were 31 non-polymorphic NNRTI-associated drug resistance mutations present on three or more expert lists (http://hivdb.stanford.edu/cgi-bin/AgMutPrev.cgi). Seventeen mutations were on five lists; seven mutations were on four lists; and seven mutations were on three lists. None of these mutations occurred at a frequency of >0.5% in any subtype.

Thirteen non-polymorphic NNRTI-resistance mutations including K101P, V179F, Y181V, and A98G (five expert lists); G190C/T/V and K103T (four expert lists); and K103H, E138K, F227C, K238T, and L318F (three expert lists) were not on the 2007 SDRM list. Five of these mutations, K101P, E138K, V179F, Y181V, and F227C have been associated with reduced susceptibility to the most recently approved NNRTI etravirine [12], [13], [14].

We added 3 of the 13 new mutations to the updated SDRM list: K101P, V179F, and Y181V. Although V179F is an uncommon mutation that occurs at a highly polymorphic position, this mutation was retained because of its frequent selection by etravirine and its profound effect on etravirine susceptibility when it occurs in combination with Y181C/I/V [15]. The mutations K103H/T, G190C/T/V and F227C were not added because they occurred at a frequency of <0.5% among ARV-experienced individuals among published sequences. A98G, E138K and K238T, although nonpolymorphic, occur at highly polymorphic positions and their prevalence among NNRTI-experienced patients is also low. P236L, which was on the 2007 list, was removed because its highest prevalence among treated persons in any subtype was 0.4% and because it is associated with resistance solely to delavirdine an NNRTI that is rarely used. G190Q, which was on the 2007 list, was removed because its highest frequency among treated individuals in any subtype was 0.1%. L318F was not added because it is not consistently sequenced during surveillance studies.

Although they occur at polymorphic postions, K101E/P, K103N/S, V106A/M, and V179F are included in the list because of their substantial contribution to resistance to commonly-used or new NNRTIs.

Several known NNRTI-resistance mutations were excluded from consideration as an SDRM because they are polymorphic in one or more subtypes including (i) V90I which occurs in 0.7%, 6.9%, 1.8%, 1.0%, 1.7%, and 0.8% of CRF01_AE, CRF02_AG, subtype B, subtype C, subtype D, and subtype G sequences, respectively; (ii) K101Q which occurs in 0.9% and 0.6% of CRF02_AG and subtype B sequences; (iii) V106I which occurs in 0.7%, 4.7%, 2.1%, 2.6%, 4.7%, and 1.6% of subtype A, CRF01_AE, subtype B, subtype D, subtype F, and subtype G isolates; (iv) V108I which occurs in 1.3%, and 0.6% of CRF02_AG and subtype B isolates; and (v) V179D which occurs in 1.7% of CRF01_AE and 4.1% of subtype F isolates; and (vi) V179E which occurs in 0.6% and 7.2% of CRF02_AG and subtype G isolates.

Table 3 contains the updated list of 19 NNRTI SDRMs at 10 RT positions. The proportion of reportedly drug-naive individuals in the dataset with one or more NNRTI-associated SDRM for each of the eight subtypes would be as follows: 0% in subtypes F and G, 0.4% in subtype A and CRF01_AE, 0.5% in subtype C, 0.5% in CRF02_AG, 0.6% in subtype D, and 0.8% in subtype B.

**Table 3:** Non-Nucleoside RT Inhibitor Surveillance Drug Resistance Mutation (SDRM) List: 19 Mutations at 10 Positions

Position	AA	Lists	New	A (%)	AE (%)	AG (%)	B (%)	C (%)	D (%)	F (%)	G (%)	No Rx (Max %)	Max Rx (%)
***Number of individuals:***				**1,305**	**770**	**1,035**	**5,672**	**2,020**	**324**	**265**	**403**	**11,784**	**14,621**
100	I	5		0	0	0	0	0	0	0	0	0	5.4
101	E	5		0	0	0	0.2	0	0	0	0	0.2	6.4
	P	5	√	0	0	0	0	0	0	0	0	0	1.4
103	N	5		0.1	0	0.2	0.3	0.2	0	0	0	0.3	40
	S	4		0	0	0	0	0	0	0	0	0	0.6
106	M	5		0	0	0	0	0	0	0	0	0	12
	A	5		0	0	0.1	0	0	0	0	0	0.1	1.1
179	F	5	√	0	0	0	0	0	0	0	0	0	0.2
181	C	5		0.1	0.1	0.1	0	0.1	0	0	0	0.1	14
	I	5		0.1	0	0	0	0	0	0	0	0.1	1.1
	V	5	√	0	0	0	0	0	0	0	0	0	1.1
188	L	5		0	0.3	0	0	0	0	0	0	0.3	2.4
	H	5		0	0	0	0.1	0	0.3	0	0	0.3	0.6
	C	5		0	0	0.1	0	0	0	0	0	0.1	0.9
190	A	5		0.1	0	0	0	0.1	0.3	0	0	0.3	10
	S	5		0	0	0	0	0	0	0	0	0	1.6
	E	4		0	0	0.1	0	0	0	0	0	0.1	0.5
225	H	5		0	0	0	0	0	0	0	0	0	7.6
230	L	3		0	0	0	0	0	0	0	0	0	3.6
***Sum of Prevalences:***				**0.4**	**0.4**	**0.6**	**0.7**	**0.5**	**0.6**	**0**	**0**		

**Abbreviations:** Pos – amino acid position; AA – amino acid difference from consensus B; Lists – Number of mutation lists with the mutation; New – mutations not present on the 2007 SDRM list; No Rx – highest prevalence in untreated persons in any of the 8 listed subtypes; Max Rx – Prevalence of the mutation in the subtype with the highest prevalence of the mutation provided the mutation is present in viruses from two or more individuals.

### PI SDRMs

There were 51 non-polymorphic PI-associated drug resistance mutations present on three or more expert lists (http://hivdb.stanford.edu/cgi-bin/AgMutPrev.cgi). Thirty-one mutations were on five lists; 12 mutations were on four lists; and eight mutations were on three lists. M46I was present in 0.6% of the isolates from 902 CRF01_AE-infected individuals, K43T was present in 0.7% of the isolates from 598 subtype F-infected individuals, and Q58E was present in 0.6% of the isolates from 515 subtype D-infected individuals.

Twenty mutations including L10F/R, K20T, L23I, K43T, M46L, G48M, F53Y, Q58E, V71I/L, T74P, L76V, V82C/L, N83D, I85V, L89V/T, and I93M were not on the 2007 SDRM list. Of these 20 mutations K43T, Q58E, T74P, V82L, and N83D have been newly recognized primarily because of their association with tipranavir resistance [16]; whereas L76V and L89V have been newly recognized primarily because of their association with darunavir resistance [17]. M46L, which had been excluded from the previous SDRM list because of its presence in 1.5% of 264 subtype G sequences, was added back to the list because its prevalence decreased to 0.5% with the approximate tripling of the number of PI-naïve sequences belonging to this subtype.

We added nine of the 20 new mutations to the updated SDRM list: L23I, M46L, G48M, F53Y, L76V, V82L/C, N83D, and I85V. Ten mutations were not added because they occur at polymorphic positions (L10F/R, K20T, K43T, V71I/L, T74P, L89V/T, and I93M). Q58E was not added because it displayed borderline polymorphism rates in multiple subtypes: 0.6% in subtype D, 0.4% in subtype B, and 0.3% in subtype C. M46I was retained despite occurring in 0.6% of 902 CRF01_AE isolates and 0.3% of subtype A and B isolates because it reduces susceptibility to several PIs even in the absence of other SDRMs [18] and because it did not occur at a frequency of >0.5% in more than one subtype with >1,000 sequences available for analysis. Examination of the CRF01_AE sequences with M46I revealed no evidence for sequence artifact or epidemiological clustering.

Although they occur at polymorphic positions, we included the protease mutations V82A/T/F/S/C/M/L because of their substantial contribution to resistance to several PIs.

Table 4 contains the updated list of 40 PI SDRMs at 18 protease positions. The proportion of reportedly drug-naive persons in the dataset having one or more PI-associated SDRMs for each of the eight subtypes would be as follows: 0.4% in subtype D, 1.0% in subtypes B and C, 1.1% in subtype A and CRF01_AG, 1.2% in subtypes F and G, and 1.5% in CRF01_AE.

**Table 4:** Protease Inhibitor (PI) Surveillance Drug Resistance Mutation (SDRM) List: 40 Mutations at 18 Positions^*^

Position	AA	New	Lists	A (%)	AE (%)	AG (%)	B (%)	C (%)	D (%)	F (%)	G (%)	No Rx (%)	Max Rx (%)
***Number of individuals:***				**1,528**	**902**	**1,437**	**7,439**	**2,182**	**515**	**598**	**619**	**15,220**	**7,886**
23	I	√	3	0.1	0.1	0	0	0	0	0	0	0.1	2.1
24	I		5	0	0	0.1	0	0	0	0.2	0	0.2	11
30	N		5	0	0	0	0	0	0	0	0	0	9.0
32	I		5	0	0	0.1	0	0	0	0	0	0.1	7.3
46	I		5	0.3	**0.6**	0.1	0.4	0.2	0	0.2	0	0.6	29
	L	√	5	0.3	0.2	0.3	0.3	0.1	0.2	0.2	0.5	0.5	13
47	V		5	0	0	0	0	0.1	0.2	0	0.3	0.3	6.8
	A		5	0	0	0	0	0	0	0	0	0	0.5
48	V		5	0	0	0	0	0	0	0	0	0	5.7
	M	√	3	0	0	0	0	0	0	0	0	0	2.3
50	V		5	0	0	0	0	0	0	0	0	0	2.5
	L		5	0	0	0	0	0	0	0	0	0	1.8
53	L		5	0	0.1	0	0	0	0	0	0.2	0.2	7.8
	Y	√	4	0.1	0	0.1	0	0	0	0	0	0.1	0.6
54	V		5	0	0	0	0	0	0	0	0	0	43
	L		5	0	0	0	0	0	0	0	0	0	4.6
	M		5	0	0	0	0	0	0	0	0	0	4.3
	A		5	0	0	0	0	0	0	0	0	0	2.6
	T		5	0.1	0	0	0	0	0	0	0	0.1	1.7
	S		5	0	0	0	0	0	0	0	0	0	1.7
73	S		5	0	0	0.1	0	0	0	0	0	0.1	13
	T		4	0	0	0	0	0	0	0	0	0	4.3
	C		3	0	0	0	0	0	0	0	0	0	2.0
	A		4	0	0	0	0	0	0	0	0	0	0.8
76	V	√	5	0	0	0	0	0.1	0	0	0	0.1	4.3
82	A		5	0	0	0	0	0.1	0	0.2	0	0.2	30
	T		5	0	0	0	0	0	0	0	0	0	13
	F		5	0	0	0	0	0	0	0	0	0	2.1
	S		5	0	0	0	0	0	0	0	0	0	1.6
	C	√	3	0	0	0	0	0	0	0	0	0	0.8
	M		4	0	0	0	0	0	0	0	0	0	2.5
	L	√	4	0	0	0	0	0	0	0	0	0	0.5
83	D	√	3	0	0	0.1	0	0.1	0	0	0	0.1	9.3
84	V		5	0	0.1	0	0	0	0	0	0	0.1	21
	A		4	0	0	0	0	0	0	0	0	0	0.8
	C		3	0	0	0	0	0	0	0	0	0	0.8
85	V	√	4	0.1	0	0.1	0.1	0.2	0	0.2	0	0.2	6.4
88	D		5	0	0.1	0.1	0.1	0	0	0	0.2	0.2	6.4
	S		5	0	0	0	0	0	0	0	0	0	3.4
90	M		5	0.1	0.3	0	0.1	0.1	0	0	0.5	0.5	45
***Sum of Prevalences:***				**1.1**	**1.5**	**1.1**	**1.0**	**1.0**	**0.4**	**1.2**	**1.2**		

**Abbreviations:** Pos – amino acid position; AA – amino acid difference from consensus B; Lists – Number of mutation lists with the mutation; New – mutations not present on the 2007 SDRM list; No Rx – highest prevalence in untreated persons in any of the 8 listed subtypes; Max Rx – Prevalence of the mutation in the subtype with the highest prevalence of the mutation provided the mutation is present in viruses from two or more individuals. Underlined bold mutations are those with a prevalence >0.5% that are nonetheless included because the >0.5% prevalence occurs in only one subtype with fewer than 1000 sequences or in fewer than 1000 sequences available for analysis.

## Discussion

The value of a standard SDRM list lies not just in obtaining an accurate estimation of transmitted resistance but also in making it possible to compare estimates of transmitted resistance from different regions and times. The SDRM list proposed in 2007 proved useful in a number of recently published surveys of transmitted ARV resistance in populations in which ARVs were recently introduced. These studies, performed in sub-Saharan Africa [19], [20], [21], [22], [23], [24] and South and South-East Asia [25], [26], reported very low levels of SDRMs consistent with the very recent introduction of ARVs into these regions.

The updated 2009 SDRM list developed in this analysis has 93 mutations including 34 NRTI-associated resistance mutations at 15 RT positions, 19 NNRTI-associated resistance mutations at 10 RT positions, and 40 PI-associated resistance mutations at 18 protease positions. The 2009 list contains 77 of the 80 mutations on the 2007 list and 16 additional mutations including four new NRTI-associated resistance mutations (D67E, K70E, L74I, and K219N), three new NNRTI-associated resistance mutations (K101P, V179F, and Y181V), and nine new PI-associated resistant mutations (L23I, M46L, G48M, F53Y, L76V, V82C/L, N83D, and I85V). Of these 16 new mutations, nine owe their recognition to specific trends in ARV development and usage: the increased use of non-thymidine containing NRTI regimens (K70E and L74I), the increased recognition of mutations associated with etravirine (K101P, V179F and Y181V), tipranavir (V82L and N83D), and darunavir (L76V) resistance. The remaining 7 mutations, owe their inclusion to the expansion in the number of mutations in the five expert system lists.

The challenge in creating an SDRM list is in choosing mutations that are both highly sensitive and specific indicators of transmitted drug resistance. Attaining a high sensitivity for transmitted resistance is challenging because the large number of HIV-1 drug-resistance mutations has the potential to make the list unwieldy. Therefore, to limit the number of SDRMs without sacrificing sensitivity, we included only established drug-resistance mutations, which with one exception (the etravirine-associated mutation V179F) occurred at a prevalence of at least 0.5% in one or more subtypes from ARV-experienced individuals.

Attaining a high specificity for transmitted resistance is challenging because many drug-resistance mutations occur naturally in untreated individuals. Although we selected only nonpolymorphic drug-resistance mutations – defined as those occurring at a prevalence ≤0.5% in untreated individuals in subtypes for which >1,000 sequences were available and at levels >0.5% in no more than one subtype for which fewer than 1,000 sequences were available – it is likely that specificity may still be compromised because “nonpolymorphic” mutations occasionally occur in the absence of selective drug pressure.

Indeed, among the 93 mutations on the 2009 SDRM list, three occurred at a frequency >0.5% (in a subtype with fewer than 1,000 sequences) and 46 occurred at a frequency between 0.1% and 0.5% in at least one subtype. The median prevalence of having one or more SDRMs in the eight subtypes in our dataset was 1.3% for the NRTIs, 0.5% for the NNRTIs, and 1.1% for the PIs. It is not possible to report an overall prevalence of sequences in our dataset having one or more mutations on the list for all three drug classes because the denominators and the individuals providing sequences for the RT and protease classes were different. However, the overall false-positive “prevalence of resistance” (based on the presence of low-level polymorphisms rather than true transmitted resistance) using our list in studies of reportedly drug-naive individuals infected with subtypes CRF02_AG, D, B, or CRF01_AE would be likely to be >3% based on the individual drug class prevalences. The use of other lists to determine transmitted resistance would result in even higher false-positive rates of transmitted resistance, because most include additional polymorphic mutations which occur at >0.5% in the absence of drug pressure.

A stricter list, which included only mutations occurring in 0% of ARV-naïve individuals in all subtypes for each drug class would eliminate this background level of drug resistance in untreated persons. However, it would be impractical, because it would eliminate many important drug resistance mutations in strains likely to be transmitted from treated individuals.

We hypothesize that the non-zero background level of mutations at many drug-resistance positions has two explanations. First several SDRMs may be genuinely polymorphic occurring at low proportions in the absence of selective drug pressure (albeit below our defined threshold of 0.5%). This low-level polymorphism may be a result of HIV-1's high error rate in combination with cytotoxic T lymphocyte (CTL) immune selection pressure. For example, the three mutations present at a frequency of >0.5% in untreated individuals – the protease mutation M46I (0.6% of CRF01_AE sequences from 902 ARV-naïve individuals) and the RT mutations M41L and M184V (1.2% and 0.6%, respectively, of subtype D sequences from 324 ARV-naïve individuals) – are each situated at terminal anchoring positions of known CTL epitopes [27] and the protease mutation M46I has been reported to disrupt recognition of the HLA-A2-restricted epitope KMIGGIGGFI encompassing protease positions 45 to 54 [28].

Second, it may be that sequences from some individuals with unreported prior treatment, or in whom resistance was transmitted, were present in our ARV-naïve dataset. Indeed, prior to the surveillance programs established by the WHO, most sequences obtained from reportedly ARV-naive individuals were from parts of the world in which ARVs had been in widespread use or were from tertiary care centers in low-income countries. In both types of study, the risk of unreported prior treatment is likely to be higher than in studies with exclusion criteria designed to limit the likelihood of including individuals with unreported previous treatment.

Experts who propose that a threshold of 2–3% be used to signal a need for undertaking resource-intensive measures to investigate or control transmission of drug-resistant HIV should consider their proposals in light of our results. Regardless of the list that is used to define mutations associated with transmitted resistance, the occurrence of polymorphic resistance-related mutations at low levels among some sequences studied is highly probable. The potential inclusion of some previously treated individuals, no matter how strict the criteria, is also possible. The confluence of minimally polymorphic drug resistance mutations and unrecognized ARV exposure may lead to falsely elevated estimates of transmitted resistance. Studies of transmitted drug resistance must be designed and interpreted with caution. Specifically, it is essential to minimize the risk that previously treated individuals are included in surveillance studies. Second, transmitted resistance should be estimated separately for specific drug classes rather than reporting an overall prevalence of “transmitted resistance”, which will multiply the effect of low-level polymorphisms. Also, the occurrence of mutations associated with a drug or drug class seldom or never used in a country or region should not be considered as strong evidence for resistance transmission.

Importantly, the SDRM list provided in this paper is not designed to be used for individual patient management; in such cases we recommend the use of other clinical based algorithms. In conclusion, the extensive mutation frequency data summarized in this paper provides a useful context in which mutation prevalence data from population-based surveillance studies of transmitted resistance can be interpreted.
